# New Blood Biomarkers for the Diagnosis of AFP-Negative Hepatocellular Carcinoma

**DOI:** 10.3389/fonc.2020.01316

**Published:** 2020-08-14

**Authors:** Ting Wang, Kun-He Zhang

**Affiliations:** Department of Gastroenterology, Jiangxi Institute of Gastroenterology & Hepatology, The First Affiliated Hospital of Nanchang University, Nanchang, China

**Keywords:** hepatocellular carcinoma, AFP-negative, diagnosis, blood biomarkers, DNA, RNA, protein

## Abstract

An early diagnosis of hepatocellular carcinoma (HCC) followed by effective treatment is currently critical for improving the prognosis and reducing the associated economic burden. Alpha-fetoprotein (AFP) is the most widely used biomarker for HCC diagnosis. Based on elevated serum AFP levels as well as typical imaging features, AFP-positive HCC (APHC) can be easily diagnosed, but AFP-negative HCC (ANHC) is not easily detected due to lack of ideal biomarkers and thus mainly reliance on imaging. Imaging for the diagnosis of ANHC is probably insufficient in sensitivity and/or specificity because most ANHC tumors are small and early-stage HCC, and it is involved in sophisticated techniques and high costs. Moreover, ANHC accounts for nearly half of HCC and exhibits a better prognosis compared with APHC. Therefore, the diagnosis of ANHC in clinical practice has been a critical issue for the early treatment and prognosis improvement of HCC. In recent years, tremendous efforts have been made to discover new biomarkers complementary to AFP for HCC diagnosis. In this review, we systematically review and discuss the recent advances of blood biomarkers for HCC diagnosis, including DNA biomarkers, RNA biomarkers, protein biomarkers, and conventional laboratory metrics, focusing on their diagnostic evaluation alone and in combination, in particular on their diagnostic performance for ANHC.

## Introduction

According to GOLOBOCAN 2018 (1), liver cancer is estimated to be the sixth most commonly diagnosed cancer and the fourth leading cause of cancer deaths globally, with 841,080 new cases and 781,631 deaths, and hepatocellular carcinoma (HCC) is the dominant histological type of liver cancer (accounting for ~75–85% of all liver cancer cases), with the highest prevalence in Asian and Eastern African countries. In addition to clinical burden, HCC also poses substantial and increasing economic burden due to healthcare expenditures; the annual cost of HCC in the USA has been estimated at more than $450 million (2). The early diagnosis and effective treatment of HCC could impact both clinical outcomes and the economic burden of HCC.

Serum alpha-fetoprotein (AFP) is by far the most widely used biomarker for HCC screening, early diagnosis, and evaluation of therapeutic efficacy and prognosis (3). However, not all HCCs secrete AFP, and AFP may be elevated in cirrhosis or hepatitis cases. A systematic review showed that the sensitivity of AFP was 41–65%, with a specificity of 80–94% when using the commonly used positive cutoff value (AFP level ≥20 ng/mL) for HCC (4). A large-scale prospective multicenter study showed that the positive rates of AFP (≥11 ng/ml as the cutoff value) were 46% (616/1338) for all HCC and 23.4% (150/641) for small HCC (<2 cm) (5). Another large multicentric survey showed that AFP-negative (<20 ng/mL) rates were found in 52% (261/502) patients with small HCCs (<3 cm), in 53.5% (51/95) patients at TNM stage I, in 48% (314/656) patients with Okuda stage 1, and in some advanced HCC patients [41.5% (24/58) at TNM stage IV and 28% (17/61) at Okuda stage 3] (6), indicating that nearly a half of HCC patients are AFP-negative, especially early and small HCCs.

The diagnosis of HCC is easy when significantly increased serum AFP levels and definitive imaging features are present. However, AFP-negative hepatic cancer (ANHC) is not as easily diagnosed, as most ANHCs are early and small HCCs, often without typical imaging characteristics. Liver nodular lesions may also have HCC-like imaging findings, making ANHC patients easily misdiagnosed (7). Due to a range of influential factors on ultrasonographic diagnosis, a systematic review, and economic analysis suggested that ultrasound should not be routinely offered to patients with ANHC (8). However, the diagnosis of ANHC is important in clinical practice, because ANHC patients have a better prognosis compared with those with AFP-positive HCC (APHC) (6, 9, 10). An et al. (11) found that the 1-, 3-, and 5-year recurrence-free survival rates were 78.1, 57.5, and 40.6% in the AFP-negative group and 61.8, 37.7, and 31.4% in the AFP-positive group, respectively, while the corresponding overall survival rates were 94.4, 83.8, and 62.3% in the AFP-negative group and 87.2, 60.0, and 36.7% in the AFP-positive group, respectively. Thus, the diagnosis of ANHC is important for the improvement of prognosis in HCC patients.

Imaging techniques are useful for HCC diagnosis. Digital subtraction angiography, dynamic contrast-enhanced magnetic resonance imaging, contrast-enhanced ultrasound, and positron-emission tomography-computed tomography were found with sensitivity of 88.2, 93.9, 88.9, and 88.9%, respectively, for the diagnosis of AFP-negative small hepatic lesions (12). However, these tools are expensive and unsuitable for screening or for a first-line diagnosis of HCC. Blood biomarkers are non-invasive, safe, convenient, economic, and easy-to-repeat tools for tumor diagnosis, and compared with imaging, biomarkers in blood can be measured repeatedly with accuracy and with a relatively rapid clinical turnaround time to monitor disease progression. In this review, we describe the clinical characteristics of ANHC and systematically review and discuss recent advances in the use of blood biomarkers for HCC diagnosis, including DNA biomarkers, RNA biomarkers, protein biomarkers and conventional laboratory metrics, and their diagnostic evaluation alone and in combination, focusing on their diagnostic performance for ANHC.

## Characteristics of ANHC

The symptoms of ANHC are generally mild and non-specific, and ANHC has better clinicopathological features compared with APHC. Compared with ANHC patients, APHC cases were more likely to feature a higher female-to-male ratio, a younger age, higher HBV-positive rate, larger tumor diameter, more cirrhosis nodules, more liver capsule invasions, higher tumor multiplicity, more carcinoma cell emboli, lower differentiation grade, later BCLC stage and TNM stage, poor Edmondson–Steiner grade, poor liver function, higher short-term recurrence, and lower overall survival and disease-free survival rates after hepatectomy or radiofrequency ablation (11, 13–18). APHC patients may need comprehensive/individualized adjuvant therapy besides surgical resection and close follow-up compared with ANHC (9). In addition, patients with ANHC at the time of diagnosis are more likely to be eligible for liver transplant (19), which may be related to the high expression of the CC genotype of mannose-binding lectin-2 gene in ANHC (20). Therefore, ANHC patients can benefit more from treatment.

The reduced malignancy of ANHC may be related to the reduced expression of related proteins, such as AFP and secretory protein c19orf10. AFP levels in HCC patients have strong relationships with unfavorable tumor features (such as histological grade, tumor size, and vascular invasion) and staging classification (9, 11, 21–23). AFP has oncogenic effects on promoting the proliferation and metastasis of HCC cells (24, 25). AFP can induce cell proliferation, migration, and invasion in ANHC (26). Overexpression of secretory protein c19orf10 can enhance ANHC cell proliferation via the activation of Akt/mitogen-activated protein kinase pathways (27). In addition, the development of ANHC is associated with DNA hydromethylation-mediated dysregulation of associated genes, such as leucine-rich repeat protein phosphatase 1 and actin-dependent regulator of chromatin, subfamily A, member 2, and associated trans-regulatory factors, such as nuclear factor I and GATA-binding protein 3, which may be a novel epigenetic regulation mechanism and potential diagnostic and prognostic biomarker of ANHC (28).

Slight differences in immunophenotypic features are also found between ANHC and APHC. CD44 positivity and higher tumor histological grade are more frequent in APHC, while nuclear beta-catenin positivity is more common in ANHC (29). In the ultrastructural morphology by transmission electron microscopy, the positive rate of Tn protein (Thomsen–Friedenreich-related antigen) was markedly higher in ANHC than in APHC, while AFP showed the opposite expression pattern; in addition, most ANHC cells were dispersed loosely with simple organelles but with abundant free polyribosomes (30); however, the APHC cells were all linked closely together and had rich organelles in their cytoplasm, especially the rough endoplasmic reticula, mitochondria, and complex Golgi, which might be related to the role of AFP in promoting cell proliferation.

## Blood Biomarkers for ANHC Diagnosis

**Figure 1:**
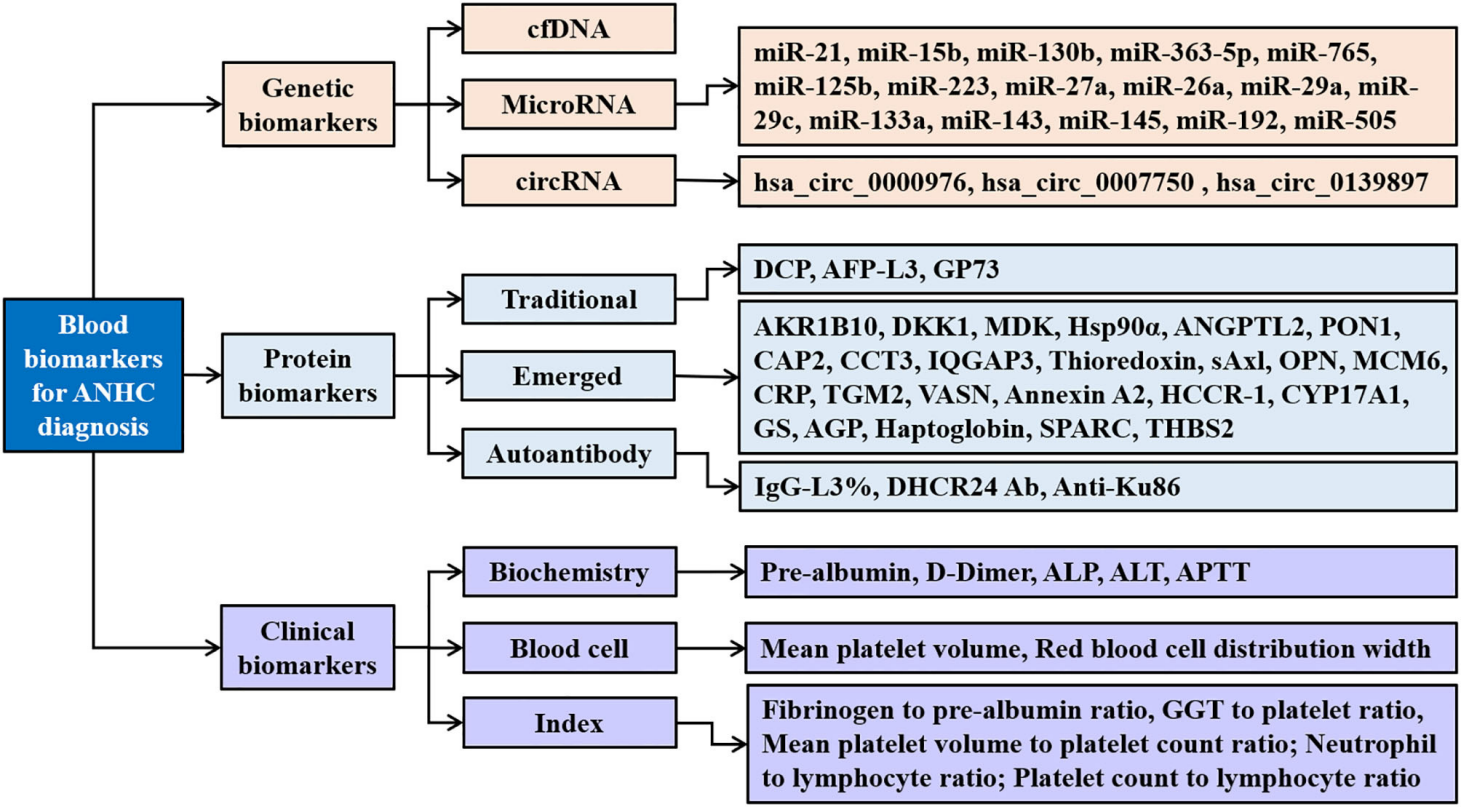
Blood biomarkers for AFP-negative hepatocellular carcinoma. ANHC, alpha-fetoprotein-negative hepatocellular carcinoma; cfDNA, circulating cell-free DNA; circRNAs, circular RNAs; DCP, des-gamma-carboxy prothrombin; AFP-L3, α-fetoprotein fraction L3; GP73, golgi protein 73; AKR1B10, aldo-keto reductase family 1 member B10; DKK1, dickkopf-1; MDK, midkine; Hsp90α, heat shock protein 90alpha; ANGPTL2, angiopoietin-like protein 2; PON1, paraoxonase 1; CAP2, cyclase-associated protein 2; CCT3, chaperonin containing TCP1 complex subunit 3; IQGAP3, IQ-motif-containing GTPase-activating protein-3; sAxl, soluble transforming receptor tyrosine kinase; OPN, osteopontin; MCM6, minichromosome maintenance complex component 6; CRP, c-reactive protein; TGM2, tissue transglutaminase 2; VASN, vasorin; HCCR-1, human cervical cancer oncogene 1; CYP17A1, the cytochrome P450, family 17, subfamily A, polypeptide 1; GS, glutamine synthetase; AGP, alpha-1 acid glycoprotein; THBS2, thrombospondin-2; IgG, immunoglobulin G; DHCR24 Ab, 3β-hydroxysterol Δ24-reductase antibody; ALP, alkaline phosphatase; ALT, alanine aminotransaminase; APTT, activated partial thromboplastin time; GGT, gamma-glutamyl transpeptidase.

The detection of disease-related molecules in blood is simple and non-invasive and is widely regarded as the best choice for disease screening and diagnosis. In recent years, many biomarkers in blood have been identified and evaluated for the diagnosis of ANHC (Figure 1), such as genetic biomarkers, proteins, and also metabolic biomarkers (31).

### Genetic Biomarkers

The development of ANHC is a chronic process and involves complicated genetic changes. Zhang et al. (28) identified 615 differentially hydroxymethylated regions from ANHC tissues compared to adjacent normal tissues, which were significantly enriched in gene ontology functions, and they found that some hydroxymethylated genes were involved in ANHC development. Lu et al. (32) found that a panel based on four candidate genes (COL5A1, HLA-DQB1, MMP2, and CDK4) was valuable for the diagnosis of ANHC patients [with an area under the receiver operating characteristic curve (AUROC) of 0.768], and Zheng et al. (33) also found that a blood-based 22-gene signature was valuable for ANHC screening (AUROC of 0.93, sensitivity of 91.3%, specificity of 83.2%). Circulating cell-free DNA (cfDNA), microRNAs, and circular RNAs are easily detected genetic biomarkers that are valuable for the diagnosis of HCC, including ANHC.

#### Circulating Cell-Free DNA

cfDNA are extracellular DNA molecules released into blood from apoptotic or necrotic cells or tissues (34). cfDNA is elevated in various malignancies, including HCC, and has cancer-specific DNA alterations, including DNA strand integrity, mutation frequency, microsatellite abnormalities, and gene methylation, and is regarded as diagnostic, prognostic, and monitoring biomarkers for cancers (35). Many methods with high sensitivity and specificity facilitate the use of cfDNA as a “liquid biopsy” for the diagnosis, prognosis, and monitoring of therapeutic response in HCC with advantages of real time and minimal invasion (36). Xiong et al. (37) found that plasma cfDNA mutations in HCC were valuable for the diagnosis of HCC, with an AUROC of 0.92, sensitivity of 65%, and specificity 100% compared with healthy controls. During the combination of cfDNA somatic mutations with AFP, they found that the AUROC was 0.96 with a sensitivity of 73% and specificity of 100% for ANHC, while for APHC, the AUROC was 0.86, with a sensitivity of 53% and a specificity of 100%. Moreover, cfDNA could predict HCC recurrence, that is, the patients with recurrent HCC showed significantly higher somatic mutation frequency of cfDNA than those without recurrence, of which the TP53 gene was the most frequently mutated gene in majority of the HCC patients (21/33, 64%).

In addition, the expression levels of cfDNA were not associated with patient age, gender, TNM stage, or AFP levels or protein induced by vitamin K absence (PIVKA-II) (38), and serum cfDNA levels could be directly, simply, and accurately detected by a real-time PCR system, which enhances their clinical application for the diagnosis of ANHC. We found that cfDNA-related fluorescence intensity and serum autofluorescence intensity were of value for the diagnosis of early, small, AFP-negative, and all primary hepatic carcinomas from liver cirrhosis (LC), chronic hepatitis, and normal control, with an AUROC value of 0.777–0.963 in the training set and 0.764–0.972 in the validation set, of which the two fluorescence intensity indicators had an AUROC of 0.836, a sensitivity of 73.6%, a specificity of 79.7%, and an accuracy of 78.6% for differentiating ANHC from non-HCC (39, 40), and their diagnostic value could be improved by combination with AFP, hepatic function tests, and/or blood cell analyses.

#### MicroRNA

MicroRNAs (miRNAs) are a family of endogenous, small (20–25 nucleotides in length), non-coding RNAs that regulate posttranscriptional gene expression by repressing messenger RNA (mRNA) translation mainly via binding at the complementary 3′-untranslated region and are well-known to play a role in human hepatocarcinogenesis. miRNAs serve as promising cancer biomarkers for diagnosis and therapy response monitoring (41), and circulating miRNAs are stable and able to be detected and quantified, which gives them either diagnostic value for an ANHC diagnosis or an additive value.

MiR-21 has been found to be dysregulated in several cancers and is associated with tumor proliferation, invasion, and metastasis (42). Serum levels of miR-21 were higher in HCC than in controls, with an AUROC of 0.849, sensitivity of 82.1%, and specificity of 83.9% for the diagnosis of HCC, which were higher than those of AFP (AUROC 0.722, sensitivity 68.7%, specificity 62.5%) (43), and the serum levels of miR-21 were also significantly related to clinical stage and distant metastasis (positive in 83.3% HCC patients) but not with AFP. MiR-21 had a positive rate of 77.6% (45/58) in the ANHC group and an AUROC of 0.831, sensitivity of 81.2%, and specificity of 83.2% for the diagnosis of ANHC (43). Additionally, the serum levels of miR-21 were significantly decreased after surgery in patients with HCC (43), indicating that miRNAs can be used for monitoring treatment response.

The combination analysis of miRNAs can complement each other and effectively improve their diagnostic performance for ANHC. A classifier established by the combination of miR-15b and miR-130b had an AUROC of 0.980 (96.7% sensitivity and 91.5% specificity) for detecting ANHC (44), and this miRNA classifier could identify 44 of 45 (97.8%) HCC cases with tumor-node-metastasis stages I and II, whereas serum AFP (cutoff value 20 ng/ml) could only detect 22 of 45 (48.9%) of the same cases; also, miR-15b and miR-130b were markedly reduced after surgery. Tian et al. (45) combined miR-363-5p and miR-765 with PIVKA-II and established a logistic regression model for predicting ANHC and found that the model had the AUROC of 0.930, sensitivity of 79.4%, and specificity of 95.4% in the testing set, higher than any single indicator, and AUC of 0.936, sensitivity of 83.6%, and specificity of 94.7% in the validation set. The combination of four miRNAs (miR-125b, miR-223, miR-27a, and miR-26a) showed an AUROC of 0.849, sensitivity of 80.0%, and specificity of 89.4% for distinguishing HBV-related AFP-negative early-stage HCC and the non-cancer subjects (46); interestingly, the panel that combined two miRNAs (miR-125b and miR-27a) also had comparable diagnostic performance to the four-miRNA panel above, with an AUROC of 0.845, sensitivity of 80.0%, and specificity of 87.2% for differentiating HBV-related early-stage ANHC from non-cancer, indicating that selecting appropriate complementary biomarkers for combined detection can not only simplify detection methods but also reduce detection costs.

MiRNAs have the ability to detect small-size, early-stage, AFP-negative HCC, which provides a chance of curative resection for HCC patients. Lin et al. (47) conducted a large-scale, multicenter nested case–control study to evaluate a seven-miRNA classifier (miR-29a, miR-29c, miR-133a, miR-143, miR-145, miR-192, and miR-505) for HCC in at-risk patients. The authors found that the miRNA classifier had an AUROC of 0.825 for detecting ANHC, with sensitivities of 78.8, 75.8, and 80.0% and specificities of 86.3, 88.2, and 91.1% in the training cohort, validation cohort 1, and validation cohort 2, respectively, and had an AUROC of 0.812 to identify ANHC from at-risk controls, and this classifier also had larger AUROCs than did AFP to detect small-size (AUROC 0.833 vs. 0.727) and early-stage (AUROC 0.824 vs. 0.754) HCC.

#### Circular RNAs

Circular RNAs (circRNAs) are covalently closed, single-stranded, and stable transcripts (48) that have been found to play important roles in the diagnosis of various cancers, including gastric cancer (49–51), breast cancer (52), lung cancer (53, 54), pancreatic cancer (55), and HCC (56, 57). A large-scale, multicenter study successfully validated three circRNAs (hsa_circ_0000976, hsa_circ_0007750, and hsa_circ_0139897) in the plasma of the hepatitis B virus-related HCC patients, and their plasma levels positively correlated with HCC tissue levels and significantly decreased after hepatectomy (58), and the CircPanel developed by using binary logistic regression based on the three circRNAs showed a significantly higher accuracy than AFP to differentiating HCC patients from controls in all three sets (AUROC, 0.863 vs. 0.790 in the training set; 0.843 vs. 0.747 in validation set 1 and 0.864 vs. 0.769 in validation set 2). The CircPanel also had a high diagnostic accuracy in the diagnosis of ANHC and AFP-negative small-HCC (all AUROCs ranged from 0.823 to 0.902, sensitivity ranged from 74.0 to 82.2%, specificity ranged from 90.6 to 97.7%) (58). Therefore, circRNAs may be potential diagnostic and therapeutic response biomarkers for HCC and ANHC.

### Protein Biomarkers for ANHC Diagnosis

#### Traditional Serum Protein Biomarkers

Several well-known traditional serum biomarkers used for the diagnosis of HCC have been explored for detecting ANHC alone or in combination (59). Prothrombin induced by vitamin K absence II (PIVKA-II), also named des-gamma-carboxy prothrombin (DCP), was found to be expressed significantly higher in early-stage HBV-related HCC than in chronic hepatitis B and to be valuable for the diagnosis of ANHC, with an AUROC of 0.73, sensitivity of 51.0%, and specificity of 84.5–94.3% (60, 61), and patients with poorly differentiated or undifferentiated HCC and microvascular invasion exhibited higher levels of PIVKA-II. Another large-scale, multicenter study found that DCP could be complementary to AFP in detecting ANHC and excluding LC with AFP positivity (62) and is also beneficial for HCC surveillance, early diagnosis, and monitoring treatment response and recurrence in the HBV-infected population, with positive rates of 63.2–76.3% in ANHC and an AUROC of 0.856, sensitivity of 76.3%, and specificity of 89.1% for the diagnosis of ANHC, and better than AFP in the surveillance of early HCC (AUROC 0.837 vs. 0.650) and discriminating HCC from LC (accuracy: 92.9 vs. 64.7%), and moreover, higher DCP levels were associated with worse clinical behaviors and shorter disease-free survival. These results indicate that serum PIVKA-II is a potential early diagnostic and prognostic biomarker for HCC, including ANHC.

Alpha-fetoprotein fraction L3 (AFP-L3) is a traditional diagnostic marker for HCC, but the conventional AFP-L3 detection methods are insufficiently sensitive in patients with low-level AFP. With a highly sensitive method, AFP-L3% had a sensitivity of 41.5% and a specificity of 85.1% for the diagnosis of ANHC (63), and the combination of AFP-L3% and DCP effectively improved the diagnostic value for ANHC (sensitivity of 44.9–90.6%). Additionally, the survival rate of patients with high AFP-L3% ratio (≥5%) was significantly lower than that of patients with low AFP-L3% (<5%) (63).

Another study (64) found that PIVKA-II was not correlated with AFP, AFP-L3, and tumor characteristics, and the combination of PIVKA-II and AFP-L3 was capable of improving HCC detection regardless of AFP levels, with an AUROC of 0.939, sensitivity of 92.1%, and specificity of 79.7% in ANHC, which was higher than that of AFP-L3 alone (AUROC 0.824, sensitivity 71.1%, and specificity 83.8%) and PIVKA-II alone (AUROC 0.774, sensitivity 57.9%, and specificity 95.9%). Moreover, the two biomarkers in combination could detect 81.8% of early-stage HCC, 86.7% of small HCC, and 91.7% of single tumor of HCC in the ANHC group (64).

As a single biomarker has insufficient sensitivity and/or specificity for the diagnosis of ANHC, a combination of multiple biomarkers is usually used to effectively improve diagnostic efficacy (65). An integrated parameter AFP/GP73 (Golgi protein 73) was created to magnify the differential diagnosis of ANHC in terms of better sensitivity and specificity (66), which had an AUROC of 0.662, sensitivity of 68.6%, and specificity of 58.8% for differentiating ANHC from LC and an AUROC of 0.796, sensitivity of 81.4%, and specificity of 70.0% for differentiating metastatic ANHC from adenocarcinomas; these values were slightly higher than those of GP73 alone (AUROC of 0.747, sensitivity of 57.7%, and specificity of 87.0%). AFP-L3 combined with GP73 was also evaluated for the diagnostic accuracy of ANHC (67): both serum AFP-L3 and GP73 had a higher positive rate in ANHC than in non-HCC patients, with respective AUROC values of 0.609 and 0.781, sensitivity of 50 and 66%, specificity of 97.5 and 96.2%, and accuracy of 79.2 and 84.6% for the diagnosis of ANHC, while the sensitivity, specificity, and accuracy achieved 40, 100, and 76.9%, respectively, when AFP-L3 and GP73 were used in combination, indicating that AFP-L3 or GP73 could be used as a biomarker for ANHC diagnosis, but their combined use does not significantly improve diagnostic performance for ANHC.

#### Emerged Serum Protein Biomarkers

Although traditional biomarkers have certain diagnostic values for HCC, none of them was ideal in clinical practice. Therefore, new biomarkers are continuing to be explored.

##### AKR1B10

Aldo-keto reductase family 1 member B10 (AKR1B10) is a novel secretory protein that is overexpressed in multiple tumors, including lung cancer, breast cancer, and colorectal cancer (68, 69), and is a potential diagnostic and prognostic biomarker for HCC (70–73). A multicenter study (74) with 1,244 participants found that serum AKR1B10 levels were significantly increased in HCC patients compared with those in non-HCC and were associated with AFP, alanine aminotransaminase, aspartate aminotransaminase, and tumor size, but not with tumor number, vascular invasion, and TNM stage, with an AUROC of 0.896, sensitivity of 72.7%, and specificity of 95.7% for the diagnosis of HCC, and these values were better than those of AFP (AUROC 0.816, sensitivity 65.1%, and specificity 88.9%), and for ANHC cases, AKR1B10 exhibited a promising diagnostic value (AUROC 0.891, sensitivity 71.2%, and specificity 92.6%), and a similar diagnostic performance was observed in AFP-negative early-stage HCC (AUROC 0.839, sensitivity 63.4%, and specificity 90.7%). Moreover, serum AKR1B10 levels dramatically decreased 1 day after surgery and returned nearly back to normal at 3 days after surgery, indicating that AKR1B10 may also be a potential diagnostic, metastasis, and/or recurrence biomarker for ANHC (74).

##### DDK1

Dickkopf-1 (DKK1) is a 266–amino acid (35-kDa) secreted glycoprotein that is expressed in a variety of human tumors, including the pancreas, stomach, liver, bile duct, breast, cervix, esophageal, and prostate (75–77) and plays a functional role in human HCC cell migration, invasion, and tumor growth (78). Serum DKK1 levels demonstrated high diagnostic and prognostic values for HCC, especially for ANHC and early-stage HCC (79, 80). A large-scale, multicenter validation study (81) noted that serum DKK1 levels were significantly higher in HCC than in chronic HBV infection, cirrhosis, and healthy controls and were valuable for differentiating ANHC from all controls, with an AUROC of 0.841, sensitivity of 70.4%, and specificity of 90.0% in the test cohort and similar results in the validation cohort (0,869, 66.7, and 87.2%, respectively) and in the test cohort (0.870, 73.1, and 90.0%, respectively). For early-stage HCC patients in the validation cohort, DDK1 also had a good diagnostic performance (AUROC 0.893, sensitivity 72.2%, and specificity 87.2%) (81). DDK1 may also be a useful biomarker to predict the therapeutic response, as its serum levels dropped after surgery (81). However, other studies showed that plasma DKK1 levels may not be valuable for diagnosing ANHC (AUROC 0.551–0.620, sensitivity 54.4–89.1%, and specificity 37.9–61.5%) (82, 83).

##### MDK

Midkine (MDK) is a heparin-binding growth factor with multiple functions, including anti-apoptotic, migratory ion-promoting, angiogenic, and antimicrobial effects and is strongly expressed during embryogenesis and most malignant tumors, but in normal adult tissues, it is weak or undetectable (84). A large-scale, multicenter validation study (85) found that serum MDK is expressed higher in HCC than in gastrointestinal malignant tumors and in non-HCC controls, and there was no significant correlation with clinicopathological features, such as histological differentiation, stage, microvascular invasion, tumor size, and serum AFP levels, and MDK had a higher sensitivity (86.9 vs. 51.9%) but similar specificity (83.9 vs. 86.3%) for HCC diagnosis compared with AFP, even in very early-stage HCC (87.1 vs. 46.7%), and in particular, MDK had an outstanding performance for distinguishing ANHC from non-HCC controls (AUROC, 0.926) and from LC (AUROC, 0.931), with sensitivity as high as 89.2%; serum MDK levels were significantly decreased in HCC patients after curative resection and re-elevated with tumor relapse. A systematic review and meta-analysis also confirmed that MDK had AUROC of 0.91, sensitivity of 88.5%, and specificity of 83.9% for detecting ANHC (86). These results indicate that MDK could be a sensitive tumor marker for diagnosis, treatment response, and recurrence in patients with HCC, including ANHC.

##### Hsp90α

Heat shock protein 90alpha (Hsp90α) is a conserved molecular chaperone that is significantly increased in various tumors and positively correlates with tumor malignancy and metastatic ability (87), and therefore, it is regarded as a potentially important target for tumor therapy. A large-scale, multicenter clinical study (88) found that plasma Hsp90α concentrations were significantly elevated in liver cancer patients, with no significant differences among different tumor types and differentiation grades, but was positively associated with tumor staging. Hsp90α was more valuable for distinguishing HCC from non-liver cancer controls than AFP, with an AUROC of 0.965, sensitivity of 93.3%, and specificity of 90.3% (for AFP, AUROC 0.887, sensitivity 61.1%, specificity 96.3%) and exhibited a remarkable discriminating performance in early-stage liver cancer (AUROC 0.963, sensitivity 91.4%, specificity 91.3%) and in ANHC (AUROC 0.971, sensitivity 93.9%, and specificity 91.3%); similar results were observed in small liver cancers. This study also found that plasma Hsp90α dropped after treatment and increased with tumor recurrence. Plasma Hsp90α may represent an effective and timely “liquid biopsy” means for the diagnosis and therapeutic efficacy of liver cancer.

##### ANGPTL2

Angiopoietin-like protein 2 (ANGPTL2) is a secretory glycoprotein involved in vascular biology, inflammation, and tumor development (89). ANGPTL2 is overexpressed in HCC tissues compared with non-cancerous liver tissues and able to promote HCC migration and invasion (90). Zhou. et al. (91) found that serum levels of ANGPTL2 is gradually elevated with the liver injury progression and reached a peak in HCC patients with chronic HBV infection, with better diagnostic performance (AUROC 0.952, sensitivity 95.2%, specificity 81.8%, and accuracy 90.7%) than AFP (AUROC 0.824, sensitivity 71.4%, specificity 95.5%, and accuracy 81.5%) for the differentiation of HCC from healthy controls. ANGPTL2 also showed good performance for the differentiation of HCC from chronic liver diseases, with an AUROC of 0.831, sensitivity of 68.3%, specificity of 87.3%, and accuracy of 80.4%, which was also better than that of AFP (AUROC 0.777, sensitivity 42.9%, specificity 93.3%, and accuracy 79.2%), for the diagnosis of ANHC, with an AUROC of 0.919 for differentiating ANHC from healthy controls and an AUROC of 0.798 (95% CI 0.710–0.886) for differentiating ANHC from high-risk controls. Thus, ANGPTL2 may be a potential diagnostic biomarker in detecting AFP-negative HBV-related HCC.

##### PON1

Abnormal protein glycosylation is involved in different diseases, especially cancers (92). Serum paraoxonase 1 (PON1) is a highly fucosylated glycoprotein in HCC compared with LC, with an AUROC of 0.892, sensitivity of 71.4%, and specificity of 94.7% in differentiating early HCC from LC (93). For differentiating AFP-negative early HCC (*n* = 20) from LC (*n* = 20), PON1 exhibited an AUROC of 0.850, sensitivity of 90.0%, specificity of 75.0%, and accuracy of 82.5% (94). Shu et al. (95) used Fuc-PON1 (the ratio of fucosylated PON1 to total serum PON1) to differentiate AFP-negative early HCC (*n* = 76) from AFP-negative LC (*n* = 76) and found that Fuc-PON1 had an AUROC of 0.78, sensitivity of 62.2%, specificity of 67.7%, and accuracy of 64.5%, while the concentration alterations of AFP-L3 and glypican-3 (GPC3) in ANHC patients were not remarkable, indicating that Fuc-PON1 is useful in the diagnosis of AFP-negative early HCC.

##### CAP2

Cyclase-associated protein 2 (CAP2), a conserved protein, takes part in the regulation of actin cytoskeleton that is involved in cellular functions, including morphogenesis, cytokinesis, and cell migration (96). CAP2 is upregulated in multiple tumors, such as breast cancer, gastric cancer, malignant melanoma (97), and it is also upregulated in early HCC and to a greater extent in advanced HCC (98). CAP2 expression in HCC correlated with tumor size, histological grade, and clinical stage, but not with plasma AFP level, HBV infection status, and patient's gender and ag, (99), and higher levels of CAP2 were found in HCC compared with cirrhosis patients, with better performance than AFP for diagnosing general HCC (AUROC 0.86 vs. 0.75, sensitivity 82.6 vs. 59.3%, specificity 79.3 vs. 83.1%) and for diagnosing early-stage HCC patients (AUROC 0.81 vs. 0.67, sensitivity 78.6 vs. 40.4%, specificity 81.4 vs. 83.1%). CAP2 had an AUROC of 0.84, sensitivity of 82.9%, and specificity of 79.6% for the detection of ANHC (*n* = 35), and for the detection of AFP-negative early HCC, CAP2 also presented a good performance (AUROC 0.80, sensitivity 80.0%, and specificity 79.6% (99). The results above suggest that CAP2 may be a potential diagnostic biomarker for ANHC.

##### CCT3 and IQGAP3

Chaperonin containing TCP1 complex subunit 3 (CCT3) is a crucial subunit in the complexes and involved in tumor cell proliferation and the tumorigenesis (100). Overexpressed CCT3 is associated with HCC progression (101, 102). IQ-motif-containing GTPase-activating protein-3 (IQGAP3) is involved in the proliferation of epithelial cells (103) and liver regeneration (104). Both CCT3 and IQGAP3 genes, localized on 1q22, were upregulated in HCC (105). Qian et al. (106) found that both plasma CCT3 and IQGAP3 levels were higher in HCC than in non-HCC, correlated well with each other (*r* = 0.824), and associated with HCC etiology, tumor size and number, and Child–Pugh classification. Plasma CCT3 and IQGAP3 were both valuable for differentiating ANHC (*n* = 38) from LC (*n* = 88) (CCT3 with an AUROC of 0.871, sensitivity of 92.1%, and specificity of 70.5% and IQGAP3 with an AUROC of 0.804, sensitivity of 81.6%, and specificity of 71.6%) and differentiating small HCC (*n* = 47) from LC (CCT3 with an AUROC of 0.761, sensitivity of 76.6%, and specificity of 70.5% and IQGAP3 with an AUROC of 0.753, sensitivity of 74.5%, and specificity of 71.6%), which were better than that of AFP (AUROC 0.707, sensitivity 53.2%, and specificity 68.2%) (106). For the diagnosis of AFP-negative small HCC (*n* = 27), CCT3 exhibited an AUROC of 0.84, sensitivity of 88.9%, and specificity of 70.5%, and IQGAP3 exhibited an AUROC of 0.822, sensitivity of 85.2%, and specificity of 71.6%) (106). The combination of AFP, CCT3, and IQGAP3 was significantly superior to AFP alone in discriminative ability (AUROC 0.954 vs. 0.815), indicating that the expression of CCT3 and IQGAP3 is independent of AFP and thus complementary to AFP for AFP-negative and small HCC diagnosis.

##### Thioredoxin

Thioredoxin is a thiol oxidoreductase that is ubiquitously expressed and is highly expressed in a variety of malignancies and associated with aggressive tumor growth and poor survival (107–109). Its expression level positively correlated with tumor size, Child–Pugh classification, or tumor stage of HCC, but not with age, sex, HBV infection time, etiology, alanine aminotransaminase, aspartate aminotransaminase, total bilirubin, prothrombin time, and AFP levels (110). Serum thioredoxin levels were significantly higher in HCC compared with chronic liver diseases and exhibited positive rates of 72.7% (40 of 55) and 69.2% (18 of 26) in ANHC and very early-stage ANHC, respectively (110). For differentiating very early HCC from non-HCC, thioredoxin had an AUROC of 0.901, sensitivity of 75.2%, and specificity of 88.9%, which were higher than that of AFP (AUROC 0.769, sensitivity 70.1%, specificity 79.4%) (110). These findings indicate that thioredoxin has the advantage over AFP for HCC detection, particularly for very early ANHC.

##### sAxl

The transforming receptor tyrosine kinase (Axl) is a member of the tumor-associated macrophage family and upregulates in several types of cancer and correlated with poor prognosis and metastasis of cancers (111, 112). The extracellular portion of Axl can be cleaved from the membrane to generate soluble Axl (sAxl) that can be detected in serum. A retrospective multicenter study found that sAxl was significantly increased in HCC compared with healthy or cirrhotic subjects and continuously elevated with the progression of HCC (113), and HCC patients with high serum sAxl levels exhibited a significantly reduced overall survival compared with low-level sAxl patients (median, 25.37 vs. 88.56 months). sAxl outperformed AFP for the detection of very early HCC (BCLC 0) (AUROC 0.848 vs. 0.797; sensitivity 76.9 vs. 38.5%), and the combination of sAxl and AFP exhibited an AUROC of 0.937, a sensitivity of 84.5%, and a specificity of 92.3% in diagnosing HCC (113). In ANHC, sAxl was also indicated as a valid diagnostic biomarker (AUROC 0.803, sensitivity 73.0%, and specificity 70.8%), and in very early ANHC, sAxl presented an even higher diagnostic value (AUROC 0.863, sensitivity 80%, and specificity 69.2%) (113). A recent study also found that sAxl had an AUROC of 0.898, sensitivity 84.6%, and specificity 76.3% for the diagnosis of ANHC and had a higher diagnostic performance (AUROC 0.881, sensitivity 94.1%, and specificity 74.2%) than that of AFP (AUROC 0.705, sensitivity 58.8%, and specificity 73.3%) for early HCC (114). These findings implicate that sAxl is a diagnostic biomarker with high accuracy for very early HCC and ANHC.

##### OPN

Osteopontin (OPN), a secreted phosphoprotein, is associated with tumor invasion, progression, or metastasis in multiple types of cancer and has been considered to be a promising target for cancer therapy (115, 116). HCC patients with elevated plasma levels of OPN were more likely to exhibit intrahepatic metastasis, early recurrence, and a worse prognosis (117). OPN was also found to be a potential biomarker complementary to AFP for HCC diagnosis. A pilot study with a small sample size found that plasma OPN levels were significantly higher in HCC patients than in cirrhosis patients, chronic hepatitis patients, and healthy controls (118), with a greater AUROC than AFP in discriminating HCC and cirrhosis patients (0.76 vs. 0.71), in discriminating early-stage HCCs and cirrhosis patients (0.73 vs. 0.68), and in discriminating ANHC and cirrhosis patients (0.75 vs. 0.59), and furthermore, in another cohort, an AUROC of 0.87 was observed for distinguishing ANHC from cirrhosis and chronic HBV. OPN was also found to be able to detect preclinical tumors, that is, 87% of patients within 2 years preceding HCC diagnosis exhibited OPN levels above cutoff value (118). Similar results were found in another study (with AUROC of 0.851, sensitivity of 79.2%, and specificity of 80.5% for diagnosing HCC and AUROC of 0.838, sensitivity of 78.3%, and specificity of 79.6% for diagnosing ANHC) (83). A meta-analysis including 8 studies (*N* = 1,399) found that serum/plasma OPN had a ability for predicting survival of HCC patients and an accuracy comparable to AFP for HCC diagnosis (the pooled sensitivity and specificity for OPN and AFP were 88 vs. 68% and 87 vs. 97%, respectively) (119); however, there is only one study to evaluate OPN for the diagnosis of early or AFP-negative HCC in this meta-analysis, so further assessment for the diagnostic value of plasma OPN in early and AFP-negative HCC is required.

##### MCM6

Minichromosome maintenance complex component 6 (MCM6) is a member of minichromosome maintenance proteins, which is indispensable for DNA replication during the initiation of S phase of the cell cycle (120). Plasma MCM6 mRNA and protein levels were significantly upregulated in HCC and correlated with vascular invasion, tumor progression, and lymph node metastasis but not with AFP levels or clinical features (age, gender, tumor size, HBV or HCV infection status, or Child–Pugh score) (121), with a sensitivity of 67.2% and a specificity of 89.8% for MCM6 protein to discriminating HCC from non-HCC. Both of MCM6 mRNA and protein were positive in 76.9% of ANHC patients and in 64.3 and 71.4% of small HCC patients, respectively (121). However, the sample size of this study is very small; hence, further studies are required to confirm the diagnostic value of MCM6 in HCC patients.

##### CRP

C-reactive protein (CRP) is a non-specific acute-phase protein produced by the liver in response to acute and chronic inflammation, and elevated CRP expression has been detected in multiple tumors and is associated with poor prognosis (122–125). Ma et al. (126) used a high-sensitivity CRP (hs-CRP) assay, which could be quantified as low as 0.04 mg/L of CRP and found that serum hs-CRP levels were significantly elevated in the HCC group compared with those in the non-HCC group and did not correlate with tumor Edmondson grade, TNM stage, or AFP status. Serum hs-CRP had a better performance than AFP (AUROC 0.903 vs. 0.824, sensitivity 84.2 vs. 74.4%, specificity 61.6 vs. 55.6%) for diagnosing HCC, and the diagnostic performance improved when the two indicators were combined (AUROC = 0.998, sensitivity = 94.1%), with similar positive rates between APHC and ANHC patients (86.9 vs. 84.6%) (126), indicating that serum hs-CRP level may be a useful diagnostic biomarker complementary to AFP for ANHC diagnosis. Another study also found that serum CRP-positive rate was significantly higher in the HCC than in the LC (64.15 vs. 37.97%) (127), and serum CRP levels were similar between ANHC and APHC patients. The combination of serum CRP with liver stiffness could be complementary to AFP in the identification of ANHC patients and help to distinguish HCC from LC.

##### TGM2

Tissue transglutaminase 2 (TGM2) is a stress-regulated protein that is associated with matrix stabilization, cell adhesion and migration, and cell death and survival (128). TGM2 in the tumor stroma can inhibit tumor growth and metastasis (129, 130). TGM2 is overexpressed in many types of cancer, including pancreatic carcinoma (131), breast cancer (132), ovarian carcinoma (133), and lung cancer (134). Interestingly, TGM2 expression in liver tissues showed an inverse correlation with serum AFP levels in HCC patients (135), and TGM2 was overexpressed in some AFP-deficient HCC cell lines (SK-HEP-1 and Bel-7402) and approximately half (17/32) of ANHC tissues but trace-expressed in APHC (3/29). Serum TGM2 levels were significantly higher in HCC patients and positively correlated with the histological grade and tumor size (135), indicating that TGM2 may be a useful histological and serologic candidate biomarker for ANHC diagnosis, although more studies are required to confirm the value of TGM2 in ANHC diagnosis.

##### VASN

Vasorin (VASN) is a secreted cell surface protein that is associated with vascular injury repair through inhibiting the TGF-β signaling pathway (136), and its overexpression in some human tumors can stimulate malignant progression and angiogenesis (137). In hepatoma, VASN is capable of promoting cell proliferation and migration and inhibiting cell apoptosis and is regarded as a promising biological treatment target for HCC. Higher VASN levels were verified in HCC serum compared with that in control cohorts, with an AUROC of 0.770, sensitivity of 69%, and specificity of 80.5% for the diagnosis of HCC; VASN was positive in 62% (23/37) of ANHC cases, indicating that VASN may be a potential biomarker for HCC diagnosis (138).

##### Annexin A2

Annexin A2 is a calcium-dependent, phospholipid-binding protein expressed on the surface of endothelial cells and most epithelial cells (139, 140). It upregulates in multiple tumors and plays various roles in tumorigenic processes, such as cell proliferation, apoptosis, migration, adhesion, invasion, and angiogenesis (141–143). Serum annexin A2 levels were significantly higher in HCC patients compared with non-HCC controls and did not correlate with gender, age, tumor size, differentiation degree, BCLC staging, and AFP levels (144), with a better performance than AFP (AUROC 0.800 vs. 0.690) for distinguishing HCC from hepatitis and cirrhosis. For early-stage HCC, annexin A2 also had a better diagnostic performance (AUROC 0.79, sensitivity 83.2%, and specificity 67.5%) compared with AFP (AUROC 0.73, sensitivity 54.7%, and specificity 81.3%), and the combination of annexin A2 with AFP improved the sensitivity and specificity up to 87.4 and 68.3% for early-stage HCC (144). Importantly, in ANHC patients (*n* = 74), annexin A2 had an AUROC of 0.77, sensitivity of 89.2%, and specificity of 58.5% (144). Thus, annexin A2 might be an important candidate biomarker for the diagnosis of ANHC and early-stage HCC.

##### HCCR-1

Human cervical cancer oncogene 1 (HCCR-1) is a novel human oncoprotein associated with human cervical cancer and upregulated in various human tumors in tumorigenesis and tumor progression (145, 146). HCCR-1 expression is high in HCC, moderate in LC, and at basal levels in normal control and chronic hepatitis, with higher detection accuracy (78.2%) than AFP (64.6%) for discrimination between HCC and LC. Serum HCCR was positive in 76.9% (40 of 52) ANHC patients (147); in addition, nine patients with metastatic lesions who were negative for AFP were positive for HCCR. However, another study showed that HCCR-1 has a positive rate of only 48.5% (63 of 130) in ANHC (148). In a multicenter prospective study (5), HCCR-1 levels did not significantly correlate with HCC clinicopathological characteristics such as age, gender, tumor size, and lymph node metastasis, but positively correlated with histological grading. Interestingly, AFP was positive in 97 of 164 (59.1%) HCCR-1-negative HCC patients, and the positive rate was up to 77.2% in combination of both AFP and HCCR-1, indicating that HCCR-1 expression is not associated with AFP levels in many HCC cases and thus HCCR-1 can complementary to AFP for ANHC diagnosis.

##### CYP17A1

The cytochrome P450, family 17, subfamily A, polypeptide 1 (CYP17A1), is a secretory protein that is overexpressed in the liver tissues of HCC model mice at both preneoplastic and neoplastic stages and in human HCC tissues compared with paired non-tumor tissues and other malignant tumors (lung cancer and prostate cancer) (149). Serum CYP17A1 exhibited better diagnostic performance than did AFP in differentiating HCC vs. healthy controls, with an AUROC of 0.91, sensitivity of 86.9%, and specificity of 76.8% for CYP17A1 and an AUROC of 0.78, sensitivity of 65.6%, and specificity of 65.6% for AFP (149). More importantly, serum CYP17A1 levels were positive in 89.1% of ANHC cases and not significantly different between ANHC and APHC (149), indicating that CYP17A1 is a promising biomarker for ANHC detection.

##### GS

Glutamine synthetase (GS) is a metabolic enzyme that catalyzes the synthesis of glutamine (a major energy source of tumor cells) and has been revealed as a sensitive and specific indicator for the development of HCC (150). Liu et al. (151) found that the serum levels of GS in HCC patients were higher compared with liver cirrhosis patients and healthy controls, and the AUROCs of GS and AFP for HCC diagnosis were 0.848 and 0.861, respectively, while the AUROC was 0.913 (sensitivity 81.9%, specificity 100%) for differentiating ANHC from healthy control, and the sensitivity and specificity achieved to 82.5 and 93% when combining GS with AFP. Those results indicate that GS may be a valuable biomarker for HCC diagnosis, especially for ANHC.

##### AGP

Alpha-1 acid glycoprotein (AGP) is an acute-phase glycoprotein synthesized mainly by hepatocytes and has different glycoforms dependent on the pathophysiological conditions (152), and multifucosylated AGP can be used as a novel biomarker for HCC (153). Liang et al. (154) found the trifucosylated N-glycan of AGP presented in HCC patients but absent in healthy controls and most cirrhosis patients and could differentiate HCC from cirrhosis with AUROCs of 0.707–0.751 in various causes of liver diseases and exhibited an AUROC of 0.709, sensitivity of 52%, and specificity of 80% for differentiating ANHC from LC. These results suggest that the AGP could serve as a potential marker for diagnosing HCC, including ANHC.

#### Serum Autoantibodies

At a relatively early stage of carcinogenesis, a small amount of tumor antigens can be produced by tumor cells and leads to the generation of autoantibodies. These autoantibodies are stable in blood circulation and remain elevated for a long time. Therefore, the detection of autoantibodies can improve the early detection of tumors that are difficult to detect directly (155). Many autoantibodies have been investigated for the early detection of ANHC, such as autoantibodies to centromere protein F and heat shock protein (HSP60), which were found to be positive in 73.6 and 79.3% cases of early-stage ANHC, respectively (156).

##### IgG-L3%

HCC-derived immunoglobulin G (IgG) and its abnormal glycosylations are related to carcinogenesis. The fraction of Lens culinaris agglutinin binding IgG (IgG-L3) among total serum IgG (IgG-L3%) was found to gradually increase from healthy volunteers, HBV carriers, and patients with LC to HCC patients, including ANHC patients, and to be more valuable than AFP for the diagnosis of HBV-related HCC (157), with an AUROC 0.835 vs. 0.718, accuracy 81.3 vs. 78.0%, sensitivity 86.7 vs. 66.7%, and specificity 77.8 vs. 85.6%, and also to be valuable for distinguishing ANHC (*n* = 123) from non-HCC (*n* = 234) (AUROC of 0.795, sensitivity of 80.5%, specificity of 70.0%) and from LC (*n* = 71) (AUROC of 0.711, sensitivity of 80.5%, specificity of 58.6%). In addition, patients with a high IgG-L3% value had a significantly lower overall survival rate than patients with low IgG-L3% value, and serum IgG-L3% values reduced after surgery and increased with recurrence. These results indicate that IgG-L3% could be a potential diagnostic and prognostic biomarker in HBV-related HCC.

##### DHCR24 Ab

Serum 3β-hydroxysterol Δ24-reductase antibody (DHCR24 Ab) is an autoimmune protein that is remarkably upregulated in HCV-infection patients and can be exploited as diagnostic biomarker for HCV-mediated HCC, but not for HBV-related diseases (158), with a higher AUROC than AFP and PIVKA-II in discriminating HCV-mediated chronic hepatitis from HCV-mediated HCC patients (0.860 vs. 0.840 and 0.780) and no correlation with serum AFP or PIVKA-II levels. It was revealed that 73.4% (58/79) of ANHC patients exhibited elevated serum DHCR24 Ab levels. Serum DHCR24 Ab may represent a potential biomarker for the diagnosis of HCV-related HCC with negative AFP.

##### Anti-Ku86

Ku86 is the regulatory region of a DNA-dependent protein kinase that is involved in multiple biological processes, including DNA double-strand break repair, recombination, telomere length maintenance, cell cycle progression, and transcriptional regulation (159). Its autoantibody, serum anti-Ku86, is significantly elevated in HCC patients compared with LC patients and decreased after surgical resection with a positive rate of 60.7% in small early-stage HCC with 90% specificity, whereas the sensitivities of AFP and PIVKA-II were 17.8 and 21.4%, respectively. Anti-Ku86 was not correlated with AFP and was positive in 61.7% (37/60) of ANHC cases (159). Therefore, the serum anti-Ku86 antibody may be a potential biomarker for the early detection of ANHC (160).

Some studies have shown low sensitivities of autoantibodies for ANHC diagnosis. The sensitivities of three autoantibodies (against nucleophosmin1, 14-3-3zeta, and mouse double minute 2 homolog proteins) for diagnosing ANHC ranged from 19.6 to 21.4%, with a specificity of 95% (161). Using a mini-array of multiple tumor-associated antigens as target antigens could enhance the detection of autoantibodies in cancer (162). In addition, a study found that the combination of autoantibodies against multiple TAAs was positive in 7 of 8 ANHC patients and in 6 of 8 small HCC patients, indicating that the combination analysis of anti-TAAs appears to be able to improve the diagnosis performance for ANHC (163).

#### New Protein Biomarkers Identified by “omics”

The proteome is a collection of all proteins in a biological sample. Tumor cells can secret proteins or shed proteins from its surface into body fluids as a source for the discovery of potential cancer biomarkers (164, 165). With the development of proteomics technology, numerous proteomic studies have been performed to examine specific protein profiles for the early detection of ANHC. Wu et al. (166) found 45 differentially changed serum protein/peptide peaks in HCC compared with LC using mass spectrometry techniques, and the most significant peak, 3,892, yielded 69.0% sensitivity, 83.3% specificity, and 80% positive predictive value in distinguishing HCC from LC and a favorable positive value for ANHC patients (6/8). He et al. (167) quantitatively screened out 24 differentially expressed proteins from patients with HBV-related ANHC, HBV without HCC, and healthy control subjects by using the combination of liquid chromatography and tandem mass spectrometry with isobaric tags for relative and absolute quantitation, of which 15 proteins were upregulated and 9 downregulated, but their diagnostic significance is to be assessed. She et al. (168) also revealed 14 abnormally expressed proteins specific to HCC by mass spectrometry and found CRP for the diagnosis of ANHC with an AUROC of 0.724, sensitivity of 73.0%, and specificity of 60.0%. Haptoglobin was identified with an AUROC of 0.763 for the diagnosis of ANHC (*n* = 49) from LC (*n* = 86) (169).

In addition to serum, tissue interstitial fluid was also used to identify differentially expressed proteins. Zhang et al. (170) found that two overexpressed extracellular matrix proteins from tissue interstitial fluid, SPARC (a glycoprotein involved in cell growth regulation through interactions with the ECM and cytokines), and thrombospondin-2 (THBS2) were valuable for HCC diagnosis. The combination of serum SPARC and THBS2 for distinguishing HCC (*n* = 44) with an AUROC of 0.97, sensitivity of 86%, and specificity of 100% and ANHC (*n* = 22) with an AUROC of 0.95, sensitivity of 91%, and specificity of 93% from healthy controls (*n* = 30) (170), and HCC patients with high THBS2 levels had significantly shorter disease-free survival and overall survival than those with low THBS2 levels, indicating that serum THBS2 could be used as a novel indicator for a poor prognosis of HCC.

### Conventional Laboratory Tests

Routine laboratory tests are a large pool of data that contain much disease-related information that can be used for the diagnosis and prognosis of diseases. Jing et al. (7) found that routine laboratory test indicators, serum pre-albumin and D-Dimer, were valuable for diagnosing ANHC, with an AUROC of 0.900, sensitivity of 90.1%, and specificity of 86.3% for pre-albumin and 0.868, sensitivity of 73.8%, and specificity of 87.1% for D-Dimer, and the combination of these two indicators provided an AUROC of 0.941, sensitivity of 85.7%, and specificity of 89.2% for the diagnosis of ANHC. Moreover, low levels of pre-albumin and high levels of D-Dimer were independent predictors of an unfavorable outcome for ANHC (7). Huang et al. (171) also found that the combination of fibrinogen to pre-albumin ratio and gamma-glutamyl transpeptidase to platelet ratio had a good ability to detect ANHC from the control group (AUROC = 0.977), AFP-negative chronic hepatitis (AUC = 0.745), and AFP-negative LC (AUC = 0.666) and possessed a larger area (0.943, 0.971) than fibrinogen to pre-albumin ratio and gamma-glutamyl transpeptidase to platelet ratio alone for differentiating small or early ANHC.

Mining the hidden information from abundant routine laboratory tests and establishing disease-predictive models can exhibit advantages of a least costly and noninvasive method. Data mining has been shown to be a successful way that automates analysis of data repositories and establishes models to make predictions, classifications, clustering, and clinical decision-making based on the core methodology called machine learning (172, 173). Best et al. (174) established a diagnostic algorithm based on age, sex, and tumor biomarkers of AFP, AFP-L3, and DCP for the diagnosis of HCC, and the model showed a sensitivity of 67.5% and specificity over 90% for diagnosing ANHC and an AUROC of 0.924, specificity of 93.3%, and sensitivity of 85.6% for diagnosing early-stage HCC.

Incorporation of some new biomarkers into a diagnostic model may be valuable to improve the diagnostic performance of the model. Wang et al. (175) found that fucosylation was elevated in HCC patients compared with cirrhotic patients and developed a diagnostic model that incorporated fucosylated kininogen with age, gender, serum alkaline phosphatase, alanine aminotransaminase levels, and AFP for predicting HCC incidence. This model has an AUROC of 0.970 and a true positive rate of 89% for detecting ANHC and early-stage HCC patients, whereas the AUROC of AFP was 0.597 with a true positive rate of 0% at a 5% false positive rate and presented better diagnostic performance compared with their previous model based on simple clinical metrics.

Conventional demographic and clinical characteristics also had been used for the diagnosis of ANHC. For a non-invasive prediction of ANHC, Luo et al. (176) developed a logistic regression model based on the combination of multiple hematological parameters including mean platelet volume, red blood cell distribution width, mean platelet volume to platelet count ratio, neutrophil/lymphocyte ratio, and platelet count/lymphocyte ratio, and this model presented superior diagnostic efficiency with an AUROC of 0.922, sensitivity of 83.0%, and specificity of 93.1%, and high diagnostic efficiency for the early diagnosis of ANHC and was confirmed in four validation sets from different hospitals, with AUROCs of 0.839–0.901, sensitivities of 78.3–87.7%, and specificities of 88.9–92.5%. We also used clinical metrics to establish a model for identifying HCC at various AFP levels in cirrhotic patients by binary logistic stepwise regression analysis (177), and the model incorporating 6 parameters (indicators of age, AFP, Na+, Cl–, alkaline phosphatase, and activated partial thromboplastin time) showed an AUROC of 0.854, 68.5% sensitivity, 86.6% specificity, and 80.0% accuracy for the identification of cirrhotic patients with ANHC.

## Summary and Conclusions

Because the diagnosis of ANHC is a challenge in clinical practice, many studies have been conducted to identify new blood biomarkers complementary to AFP for the diagnosis of HCC, including ANHC. The new blood biomarkers with potential value for ANHC diagnosis are summarized in Table 1. These new blood biomarkers consist of three types: DNA, RNA, and protein. Although these new biomarkers appear valuable for ANHC diagnosis, the results were usually obtained from monometer, preclinical studies with small sample sizes; therefore, further assessment in studies with large sample sizes, multiple centers, and a more rigorous design should be performed to validate the clinical diagnostic value of these biomarkers. A single biomarker alone is usually insufficient in sensitivity and specificity for the clinical detection of ANHC. The combination of several biomarkers including clinical variables could enhance the diagnostic performance for ANHC detection; thus, the development and validation of diagnostic models may be a promising approach to achieve a high efficiency for ANHC diagnosis. Conclusively, it remains a challenge to diagnose ANHC using blood biomarkers, and continuous efforts should be made in discovering new biomarkers, validating current biomarkers, and incorporating multiple biomarkers.

**Table 1:** New blood biomarkers with potential value for AFP-negative hepatocellular carcinoma diagnosis.

**Biomarkers**	**Molecule type**	**Method**	**Subject number**	**Diagnostic performance**			**References**
				**AUROC**	**Sensitivity (%)**	**Specificity (%)**	
Mutations of circulating cell-free DNA	DNA	Next-generation sequencing	Cases: 33 Controls: 37	0.960	73.0	100	(37)
Circulating cell-free DNA	DNA	Fluorescence intensity measurement	Cases: 193 Controls: 876	0.836	73.6	79.7	(39)
miR-21	RNA	Quantitative RT-PCR	Cases: 58 Controls: 278	0.831	81.2	83.2	(43)
miR-15b and miR-130b classifier	RNA	Qpcr	Cases: 30 Controls: 59	0.980	96.7	91.5	(44)
miR-363-5p, miR-765 with PIVKA-II	RNA+ protein	Qrt-PCR+ ELISA	Cases: 214 Controls: 410	0.930	79.4	95.4	(45)
miR-125b and miR-27a	RNA	Qrt-PCR	Cases: 38 Controls: 48	0.845	80.0	87.2	(46)
miRNA classifier (miR-29a, miR-29c, miR-133a, miR-143, miR-145, miR-192, and miR-505)	RNA	Qpcr	Cases: 66 Controls: 199	0·825	75.8	88.2	(47)
CircPanel (hsa_circ_0000976, hsa_circ_0007750, and hsa_circ_0139897)	circRNAs	Qpcr	Cases: UK Controls: 236	0.851	83.0	87.3	(58)
DCP/PIVKA-II	Protein	ECLIA CLEIA	Cases: 76 Controls: 285	0.856	76.3	89.1	(62)
AFP-L3	Protein	Microchip capillary electrophoresis and liquid-phase binding assay	Cases: 270 Controls: 396	0.707	41.5	85.1	(63)
AFP-L3+ PIVKA-II	Protein	Microchip capillary electrophoresis and liquid-phase binding assay	Cases: 38 Controls: 74	0.939	92.1	79.7	(64)
AKR1B10	Protein	Time-resolved fluorescent kit	Cases: 73 Controls: 280	0.891	71.2	92.6	(74)
DKK1	Glycoprotein	ELISA	Cases: 179 Controls: 407	0·841	70·4	90·0	(81)
MDK	Protein	ELISA	Cases: 121 Controls: 455	0.926	89.2	UK	(85)
Hsp90α	Protein	ELISA	Cases: 197 Controls: 743	0.971	93.9	91.3	(88)
ANGPTL2	Glycoprotein	ELISA	Cases: 30 Controls: 35	0.919	NA	NA	(91)
CAP2	Protein	ELISA	Cases: 35 Controls: 49	0.840	82.9	79.6	(99)
CCT3	Protein	ELISA	Cases: 38 Controls: 88	0.871	92.1	70.5	(106)
IQGAP3	Protein	ELISA	Cases: 38 Controls: 88	0.804	81.6	71.6	(106)
Soluble Axl	Protein	ELISA	Cases: 137 Controls: 65	0.803	73.0	70.8	(113)
OPN	Phosphoprotein	ELISA	Cases: 20 Controls: 23	0.870	AN	NA	(118)
MCM6	Protein	ELISA	Cases: 13 Controls: 59	0.857	76.9	89.8	(121)
CRP	Protein	Laser nephelometry	Cases: 65 Controls: 64	UK	95.9	92.2	(126)
Annexin A2	Protein	ELISA	Cases: 74 Controls: 123	0.770	89.2	58.5	(144)
CYP17A1	Protein	ELISA	Cases: 267 Controls: 366	NA	89.1	NA	(149)
GS	Protein	ELISA	Cases: 75 Controls: 57	0.913	81.9	100	(151)
AGP	glycoprotein	ELISA	Cases: 44 Controls: 58	0.709	52.0	80.0	(154)
Pre-albumin	Protein	Turbidimetry	Cases: 214 Controls: 210	0.900	90.1	86.3	(7)
D-Dimer	Protein	Immunoturbidimetry	Cases: 214 Controls: 210	0.868	73.8	87.1	(7)

*AUROC, area under the receiver operating characteristic curve; DCP, des-gamma-carboxy prothrombin; PIVKA-II, prothrombin induced by vitamin K absence II; AFP-L3, α-fetoprotein fraction L3; AKR1B10, aldo-keto reductase family 1 member B10; DKK1, dickkopf-1; MDK, midkine; Hsp90α, heat shock protein 90alpha; ANGPTL2, angiopoietin-like protein 2; PON1, paraoxonase 1; CAP2, cyclase-associated protein 2; CCT3, chaperonin containing TCP1 complex subunit 3; IQGAP3, IQ-motif-containing GTPase-activating protein-3; Axl, the transforming receptor tyrosine kinase; OPN, osteopontin; MCM6, minichromosome maintenance complex component 6; CRP, c-reactive protein; TGM2, tissue transglutaminase 2; CYP17A1, the cytochrome P450, family 17, subfamily A, polypeptide 1; GS, glutamine synthetase; AGP, alpha-1 acid glycoprotein; UK, unknown; qPCR, real-time quantitative PCR; qRT-PCR, quantitative real-time polymerase chain reaction; ECLIA, electrochemiluminescence immunoassay; CLEIA, chemiluminescence enzyme immunoassay*.

## Author Contributions

TW wrote the initial draft. K-HZ revised the review. All authors checked and approved the final version.

## Conflict of Interest

The authors declare that the research was conducted in the absence of any commercial or financial relationships that could be construed as a potential conflict of interest.
